# Focal Length Affects Depicted Shape and Perception of Facial Images

**DOI:** 10.1371/journal.pone.0149313

**Published:** 2016-02-19

**Authors:** Vít Třebický, Jitka Fialová, Karel Kleisner, Jan Havlíček

**Affiliations:** 1 Faculty of Science, Charles University, Prague, Czech Republic; 2 National Institute of Mental Health, Klecany, Czech Republic; Middlesex University London, UNITED KINGDOM

## Abstract

Static photographs are currently the most often employed stimuli in research on social perception. The method of photograph acquisition might affect the depicted subject’s facial appearance and thus also the impression of such stimuli. An important factor influencing the resulting photograph is focal length, as different focal lengths produce various levels of image distortion. Here we tested whether different focal lengths (50, 85, 105 mm) affect depicted shape and perception of female and male faces. We collected three portrait photographs of 45 (22 females, 23 males) participants under standardized conditions and camera setting varying only in the focal length. Subsequently, the three photographs from each individual were shown on screen in a randomized order using a 3-alternative forced-choice paradigm. The images were judged for attractiveness, dominance, and femininity/masculinity by 369 raters (193 females, 176 males). Facial width-to-height ratio (fWHR) was measured from each photograph and overall facial shape was analysed employing geometric morphometric methods (GMM). Our results showed that photographs taken with 50 mm focal length were rated as significantly less feminine/masculine, attractive, and dominant compared to the images taken with longer focal lengths. Further, shorter focal lengths produced faces with smaller fWHR. Subsequent GMM revealed focal length significantly affected overall facial shape of the photographed subjects. Thus methodology of photograph acquisition, focal length in this case, can significantly affect results of studies using photographic stimuli perhaps due to different levels of perspective distortion that influence shapes and proportions of morphological traits.

## Introduction

Human face research has received immense attention in fields ranging from social psychology to behavioural neuroscience and economics. Studies on perception of human faces involve various social contexts such as mate choice, cooperation, and parental care [1]. These studies have many practical implications; for instance, it has been shown that facial appearance affects electoral success, career progress, and child treatment [2]. Further, perceived characteristics are frequently associated with facial appearance [3]. For instance, it was recently found that facial width-to-height ratio (fWHR) is related to perceived aggressiveness [4–6], dominance [7,8], and trustworthiness [9]. Moreover, perceptions of certain psychological characteristics such as dominance [10], intelligence [11], and aggressiveness [12] can be, to some extent, predicted from overall facial morphology as analysed by geometric morphometrics.

The majority of studies on facial perception employ portrait photographs as stimuli. The facial images can be either obtained from free access sources [12–16] or taken for the purpose of the specific study [17–20]. There are several major factors affecting the resulting stimuli including exposure (e.g., under- or overexposure of images or inappropriate depth of field), optical aberrations of the lens used (e.g., radial and perspective distortions) [21,22], colour representations and lighting set up (e.g., number and type of lights and light modifier used) [23]. For instance, recent studies reported that variation in colours of facial photographs affects assessments of health, attractiveness, aggressiveness, and dominance [20,24]. Similarly, the position and posture of the target during image acquisition influences fWHR as measured from the photograph [25] or perceived body size [26]. Although several previous studies have reported carefully the standardized procedures for photograph acquisition (e.g., [27,28], employed methodology varies across individual studies. Further, detailed descriptions of photograph acquisition and standardization within a study is often missing which may impede assessment of validity and accurate replication of previous findings.

A key factor affecting the resulting photographs, which is frequently not reported in previous studies, is focal length. Focal length represents the distance between lens optics and camera sensor and provides variance in viewing angle and zoom (from wide angle fish eye lens to narrow telephoto lens) resulting in different degrees of image distortion. The most common image distortions are radial distortions, where straight lines are rendered as curved lines (i.e., barrel and pincushion distortion) [21], and perspective distortions, which are determined by the viewpoint from which the photograph is taken in relation to the target (i.e., nearby elements are rendered larger than distant ones) [22]. Due to these distortions, artefacts in size and shape representations in photographs can occur.

Here, we investigated the possible influence of focal length on perception of facial images through the assessment of selected interpersonal characteristics. Further, we tested the potential effect of focal length (50 mm, 85 mm, 105 mm) on depicted facial shape by measuring facial width-to-height ratio and employing geometric morphometrics.

## Methods

### Ethics statement

The study was approved by the Institutional Review Board of Charles University, Faculty of Science (approval number 2013/11). All participants gave written informed consent prior to taking part in the study.

### Participants

#### Targets

In total we obtained facial images of 23 target men (mean age = 23.83, SD = 4.38) and 22 target women (mean age = 22.91, SD = 4.38). The targets were recruited via social networks (e.g., Facebook) and advertisement at a part-time jobs webpage (www.jobs.cz) and were reimbursed with 100 CZK (approximately €4).

#### Raters

We recruited 369 students (176 males) from Charles University in Prague to rate a set of photographs on one of the selected characteristics (i.e., attractiveness, dominance, and femininity/masculinity). Attractiveness of the male photographs was assessed by 25 male (mean age = 21.72, SD = 2.42) and 30 female raters (mean age = 21.2, SD = 1.34). Attractiveness of the female photos was assessed by 30 male (mean age = 23.5, SD = 3.45) and 34 female raters (mean age = 22.47, SD = 3.08). Dominance of the male photographs was assessed by 30 male (mean age = 23.33, SD = 2.85) and 34 female raters (mean age = 22.53, SD = 2.08). Dominance of the female photos was assessed by 27 male (mean age = 21.85, SD = 3.19) and 30 female raters (mean age = 21.53, SD = 1.96). Masculinity of the male photos was assessed by 30 male raters (mean age = 22.97, SD = 3.01) and 33 female raters (mean age = 22.61, SD = 2.38). Finally, femininity of the female photos was assessed by 34 male (mean age = 22.56, SD = 2.35) and 32 female raters (mean age = 22.31, SD = 2.07). Raters were not reimbursed for their participation.

### Photograph acquisition

Three facial photographs of each target varying in focal length (50 mm, 85 mm, 105 mm) were taken using a DSLR camera (Nikon D90) equipped with APS-C sensor (crop factor 1.5×). Crop factor indicates how many times smaller the sensor is compared to the full frame (FF) sensor (the size of a full 35mm analogue film frame). Exposure was set to ISO 100, shutter speed 1/100s, aperture F8 and 2/3 of strobe power. We used ISO 100 to maintain the highest image quality and the lowest amount of digital noise, and shutter speed 1/100s to freeze potential motion while giving enough time for synchronizing the shutter curtains with the strobe lights [29,30]. Aperture F8 was selected to obtain the sharpest possible results with the lens used (see below) and give sufficient depth of field, i.e., the target’s face from nose tip to ear ridges was sharp without needing to change settings while taking each photograph from different distances (different distances produce variance in the depth of field). The focusing point was set on the left eye in AF-S mode. White balance was set manually using a grey background to ensure constant colour representation. Photographs were processed into JPEG files in the camera with the Nikon STANDARD colour scheme.

All images were taken from a tripod (Velbon Sherpa) with the height set for each photograph depending on the height of the target, keeping the target´s face in middle of the frame. Similarly, the distance between the camera and the target was individually adjusted for each shot so that the head of the target filled the same portion of the frame (using grid lines in live view mode) [31,32].

To test the effect of the focal length we selected the closest FF equivalents of 50 mm, 85 mm, and 105 mm focal lengths. The 85 mm and 105 mm focal lengths are the most frequently used for portrait photography [30] and do not exceed ordinary space requirements between the target and the camera, concurrently giving the same field of view. Moreover, 50 mm lenses are frequently used as standard or prime lenses, as they are believed to be equivalent in focal length to the human eye and are adept at creating natural-looking images (e.g., [21]). However, to our knowledge, it appears that there is no solid evidence supporting this notion. We used a zoom type lens Nikon AF-S DX Zoom Nikkor 18–135 mm f/3.5–5.6 G IF-ED, which allowed us to set all three focal lengths without needing to switch lenses. To use the closest FF equivalent focal length on an APS-C sensor camera, one needs to multiply the focal length stated on the given lens barrel by the crop factor of a given camera (e.g., 1.5× for Nikon APS-C). The closest possible focal lengths were: 32 mm as 50 mm FF equiv. 48 mm; 56 mm as 85 mm FF equiv. 84 mm; and 70 mm as 105 mm FF equiv. 105 mm, respectively.

**Fig 1:**
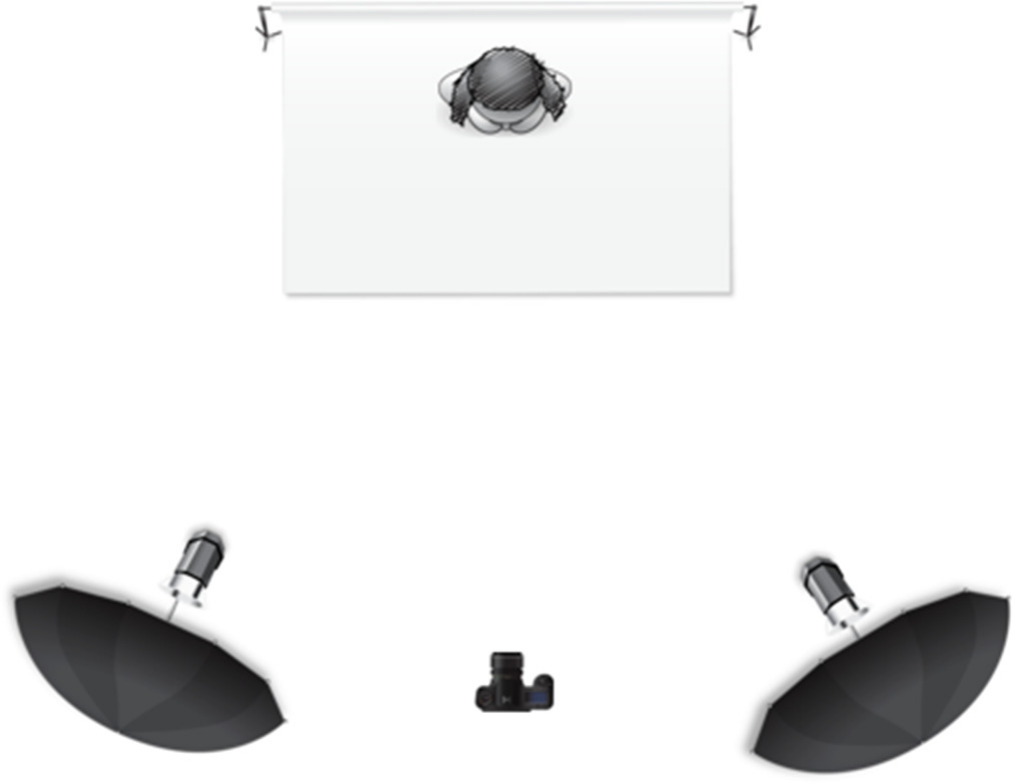
Lighting setup diagram. Visualisation was generated by the online free service Sylights.com.

The targets were asked to stand 1.5 m from a plain grey background (Storm Gray, BD Company). Two studio strobes (Menik MD300, 300W, GN 54) with white reflective umbrellas (ø102 cm) as light modifiers were used to illuminate the targets. Lights were arranged in 40° angles on each side and were 1.85 m from the target, 1.8 m height and at 35° angle incline giving even illumination (Fig 1). Light conditions in the room were controlled by the use of non-translucent curtains and all ambient lights were switched off to remove any additional lighting variables.

The targets were instructed to remove any facial cosmetics and adornments (e.g., jewellery or glasses) and their hair was pulled back from the face and held by a headband. We instructed them to keep a “neutral” facial expression, and look directly into the camera. In case of targets´ head not facing the camera directly, they were further instructed to adjust their head position accordingly (e.g., to move their chin up or down). All photographs were subsequently post-produced regarding the position of the face in the image (same position of the eyes in vertical and horizontal axis) using image manipulation software GIMP, ver. 2.8.

### Rating sessions

Rating sessions were held in a lecture room equipped with 25 identical desktop computers and 22” DELL P2210 LCD screens (set on 50% level of contrast and brightness) under standardized light conditions. Qualtrics survey building engine (www.qualtrics.com) was used to present photographs in full screen mode. Photographs were presented in a randomized order using a forced-choice test, where raters were simultaneously shown all three photographs (taken with different focal lengths) of each target. The sides of the three photographs were fully randomized (i.e., different for each target for each rater). Photographs were judged for their attractiveness, dominance, and femininity/masculinity, characteristics frequently used in facial research (for recent reviews see [33,34]). We asked raters to order the triads of the photographs with respect to given characteristic (e.g., 1 –the least attractive, 2 –medium attractive, 3 –the most attractive). Each rater assessed photographs of all 45 targets.

### Facial width-to-height ratio measurements

To investigate whether the ratio between selected facial features differs depending on the focal length used, bizygomatic width (facial width), and distance between the upper lip and brow (facial height) [4,5] was measured using tpsDig2 software, ver. 2.14 [35]. The fWHR was then calculated by dividing the facial width by the facial height. Measurements of fWHR were calculated by two independent experimenters (JF and VT). Inter-rater agreement for fWHR in images varying in focal length varied between Cronbach’s α = 0.963–0.972, indicating excellent reliability [36].

### Geometric morphometrics

Geometric morphometrics were applied to examine whether different focal lengths affect facial morphology in portrait photographs.

The 82 landmarks (including 42 semi-landmarks) were digitized by tpsDig2 software, ver. 2.14 [35]. Landmarks are represented as points that are anatomically (or geometrically) homologous in different individuals, while semi-landmarks serve to denote curves and outlines. The definitions of landmark and semi-landmark locations on human faces were based on previous work [11,12,37]. Semi-landmarks were slid by tpsRelw (ver. 1.49) software. All configurations of landmarks and semi-landmarks were superimposed by Generalized Procrustes Analysis (GPA), implemented in tpsRelw, ver. 1.53 [35]. This procedure standardized the size of the objects and optimized their rotation and translation so that the distances between corresponding landmarks were minimized. Subsequently, to visualize the effect of distortion produced by focal lengths, the original photographs of men and women were unwarped to consensual configuration of a particular focal length. The composite images were generated by tpsSuper 1.14 [38].

### Statistical analysis

To assess differences between judgements given to the images taken with varying focal lengths, we calculated the proportion (from 0 to 1) of the images taken with given focal length selected as the most attractive/dominant/masculine-feminine. As the judgements were collected using a forced-choice paradigm, they are not independent and only data on the first choice (e.g., the most attractive) were analysed. The proportion for each judgement type was then compared to a random distribution (equal to 0.333) using a one-sample Wilcoxon Signed Rank Test.

Differences in mean proportions of the first choice between images taken with individual focal lengths were compared using Kruskal-Wallis H test. Subsequently, we used Mann-Whitney U tests as post-hoc tests to compare each pair of focal lengths (50 mm × 85 mm; 50 mm × 105 mm; 85 mm × 105 mm). Bonferroni adjustment was used to correct for the effect of multiple comparisons in the Mann-Whitney U tests, with a threshold for significance of 0.017 (i.e., 0.05/3) [39,40]. Effect sizes are reported in the form of Cohen’s *d*.

A repeated measures ANOVA was employed to investigate differences in fWHR between images taken with different focal lengths. Overall effect size is reported in form of partial eta squared (η^2^), effect sizes of subsequent Bonferroni post-hoc tests are again reported in the form of Cohen’s *d*.

To test for shape differences that resulted from using various focal lengths, we performed permutational multivariate analysis of variance using distance matrices with 9,999 permutations (the Adonis function in the Vegan package in R [41]); the Euclidean method was used as a distance measure and the parameter ‘strata’ was set to constraint permutation within the groups of landmark configuration of the same photographed subject. We ran a multiple multivariate regression with principal component scores as the response variable and with focal length as an explanatory variable. Effect sizes are reported as R2.

All analyses were performed using IBM SPSS ver. 22 and R software for statistical computing [42].

## Results

### Perceived characteristics

To test for the potentially confounding effect of rater sex on the assessed characteristics, we performed an independent sample t-test. No significant differences between men and women were found on any of the rated characteristics for female or male faces: female attractiveness t_(190)_ = 0.023, p = 0.982; female dominance t_(169)_ = 0.365, p = 0.715; female femininity t_(196)_ < 0.001, p = 1; male attractiveness t_(163)_ = 0.395, p = 0.694; male dominance t_(187)_ = 0.039, p = 0.969; male masculinity t_(187)_ < 0.001, p = 1.

**Fig 2:**
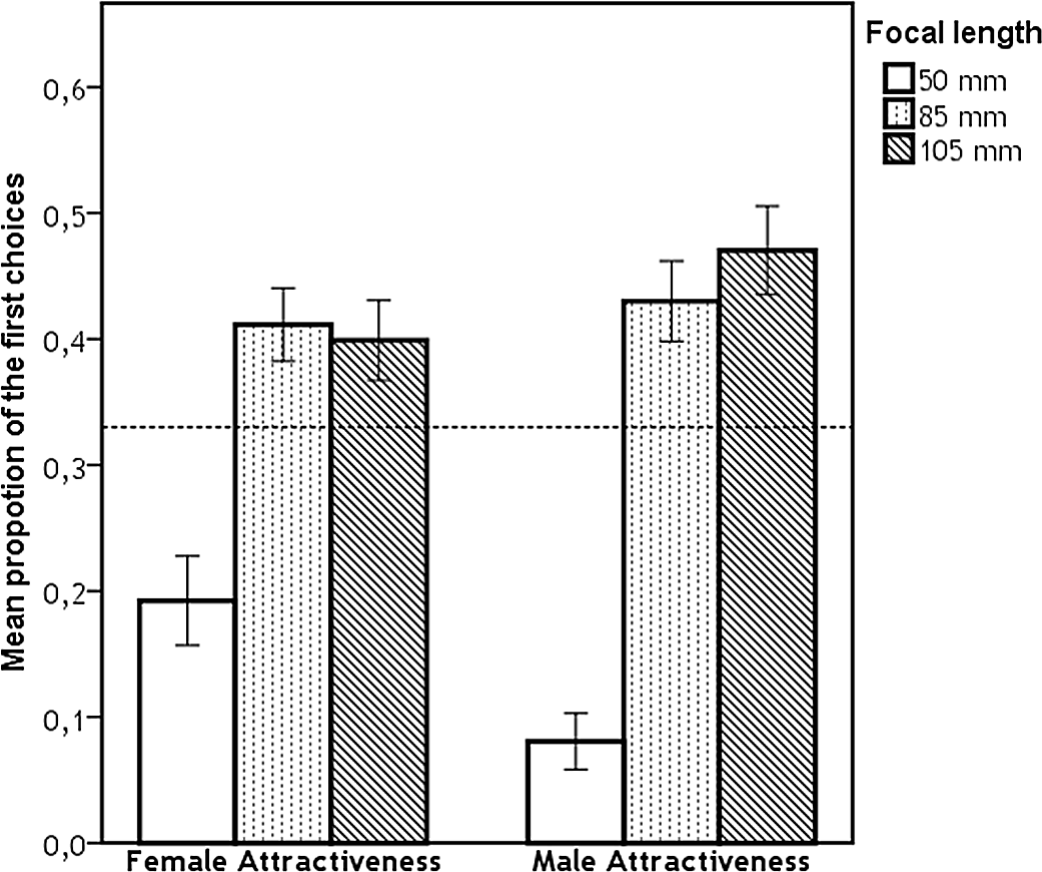
Proportion of first choices for each focal length with respect to their attractiveness. Dashed line represents the proportion of ratings expected by chance (i.e., 0.333), error bars represent 95% CI.

**Fig 3:**
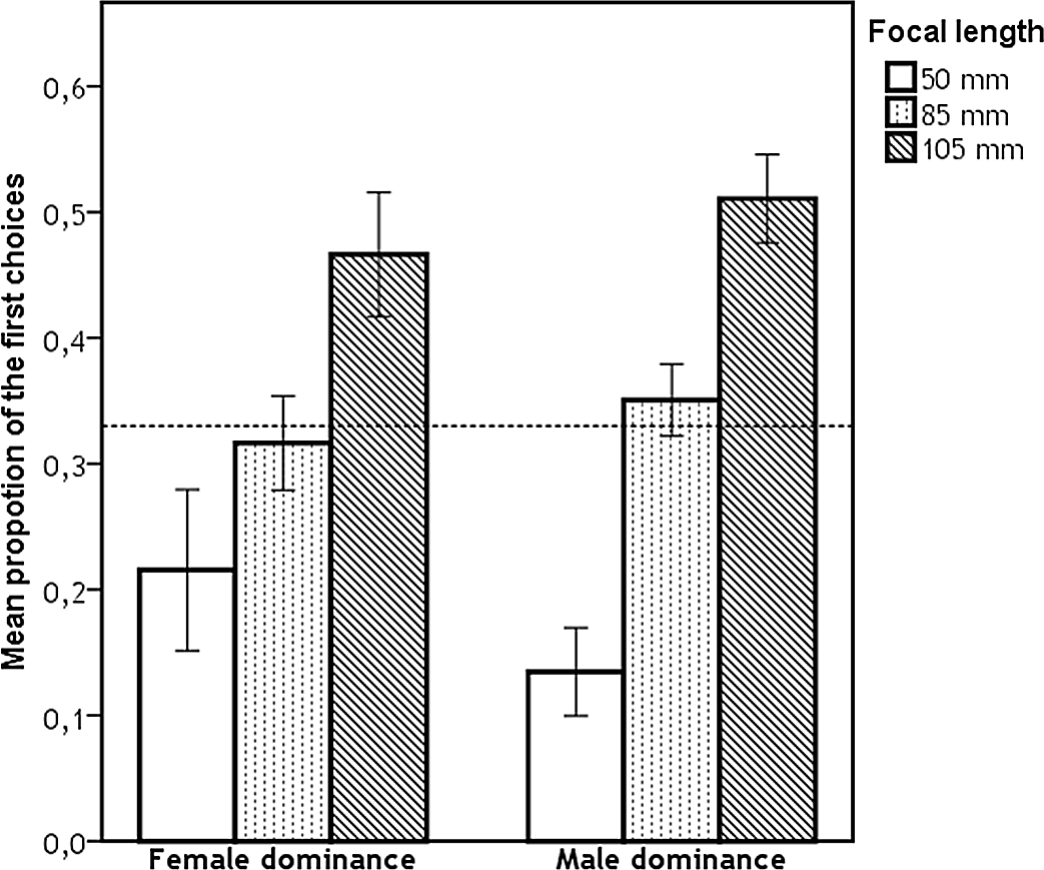
Proportion of first choices for each focal length with respect to their dominance. Dashed line represents the proportion of ratings expected by chance (i.e., 0.333), error bars represent 95% CI.

**Fig 4:**
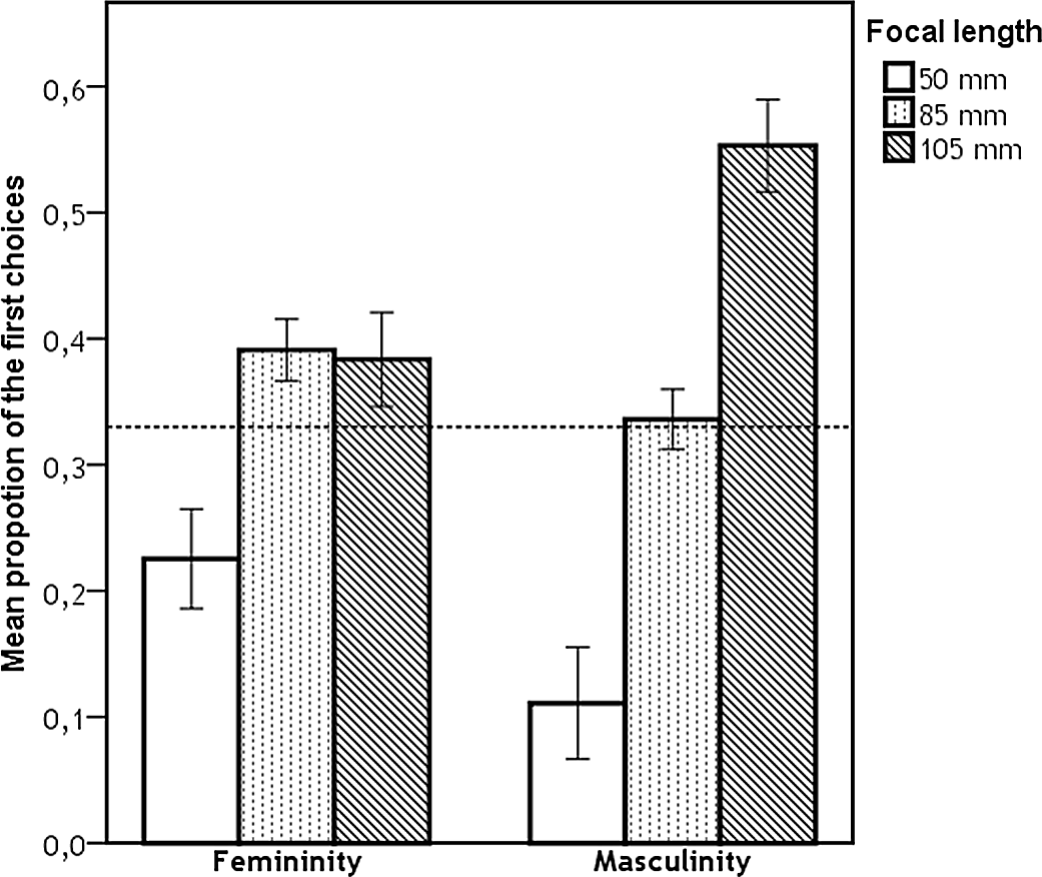
Proportion of first choices for each focal length with respect to their femininity/masculinity. Dashed line represents the proportion of ratings expected by chance (i.e., 0.333), error bars represent 95% CI.

Subsequently, we compared the proportion of the first choices for each focal length against random distribution for each type of judgement, for results see Table 1. The differences in mean proportion of the first choices (for each characteristic) between focal lengths were analysed using Mann-Whitney tests (Table 2). We found significant effect of focal length on judgements of all characteristics for both target sexes. Figs 2, 3 and 4 represent the proportion of the first choices for each focal length with respect to their attractiveness, dominance, and femininity/masculinity, respectively. More specifically, the mean proportion of the first choices given to images taken with 50 mm focal length was the lowest for all assessed characteristics and significantly differed from both 85 mm and 105 mm focal lengths. The mean proportion of the first choices given to images taken with 105 mm focal length was the highest; however, the mean proportion of the first choices given to images taken with 105 mm focal length did not significantly differ from those given to images taken with 85 mm focal length for female attractiveness, female dominance, and male attractiveness ratings.

**Table 1:** Comparison of the proportion of first choices for each focal length against random distribution (i.e., 0.333).

		One-Sample Wilcoxon Signed Rank Test	
	Focal length (mm)	Z	p
**Female attractiveness**	50	207	< 0.001
	85	1 684	< 0.001
	105	1 567	< 0.001
**Female dominance**	50	275	< 0.001
	85	751	0.548
	105	1 507	< 0.001
**Female femininity**	50	392	< 0.001
	85	1 768	< 0.001
	105	1 519	< 0.008
**Male attractiveness**	50	3	< 0.001
	85	1 351	< 0.001
	105	1 424	< 0.001
**Male dominance**	50	76	< 0.001
	85	1 130	0.403
	105	1 956	< 0.001
**Male masculinity**	50	173	< 0.001
	85	1 057	0.737
	105	1 942	< 0.001

**Table 2:** The differences in mean proportion of first choices for attractiveness, dominance and femininity/masculinity between focal lengths.

				Kruskal-Wallis H Test*		Mann-Whitney *U* test								
	Focal length (mm)	M	SD			50×85			50×105			85×105		
				χ^2^	p	*U*	p	*d*	*U*	p	*d*	*U*	p	*d*
**Female attractiveness**	50	0.1922	0.1419	69.114	<0.001	517.5	<0.001	1.696	582	<0.001	1.578	1874.5	0.405	0.148
	85	0.4099	0.1149											
	105	0.3969	0.1262											
**Female dominance**	50	0.2288	0.1429	58.25	<0.001	948	<0.001	0.775	413	<0.001	1.689	724	<0.001	1.095
	85	0.3261	0.1242											
	105	0.4577	0.1338											
**Female femininity**	50	0.2253	0.1607	45.158	<0.001	816	<0.001	1.288	1002.5	<0.001	1.056	2100.5	0.723	0.062
	85	0.391	0.0994											
	105	0.3835	0.1521											
**Male attractiveness**	50	0.1345	0.1396	100.737	<0.001	74.5	<0.001	2.912	78	<0.001	2.885	1208.5	0.067	0.354
	85	0.3505	0.1131											
	105	0.5106	0.1392											
**Male dominance**	50	0.1108	0.1766	107.739	<0.001	516	<0.001	1.669	154	<0.001	2.647	771.5	<0.001	1.248
	85	0.3359	0.0945											
	105	0.5531	0.145											
**Male masculinity**	50	0.0806	0.0821	116.152	<0.001	438	<0.001	1.836	227.5	<0.001	2.400	444.5	<0.001	1.814
	85	0.43	0.1176											
	105	0.4703	0.1301											

* df = 2 for all comparisons

### Facial width-to-height ratio

Repeated measure ANOVA showed that fWHR in female targets significantly varied across the individual focal lengths (F_(2,42)_ = 120.511, p < 0.001, η^2^ = 0.852). Subsequent Bonferroni post-hoc tests revealed significant differences between 50 mm (mean = 1.716, SD = 0.023) and 85 mm (mean = 1.797, SD = 0.024) (p < 0.001, *d* = 3.446), 50 mm and 105 mm (mean = 1.1797, SD = 0.022) (p < 0.001, *d* = 3.599), but not between 85 mm and 105 mm (p = 1, *d* = 0).

In a similar fashion, fWHR in males also significantly varied across the individual focal lengths (F_(2,44)_ = 176.419, p < 0.001, η^2^ = 0.889). Bonferroni post-hoc tests revealed significant differences between 50 mm (mean = 1.695, SD = 0.024) and 85 mm (mean = 1.787, SD = 0.026) (p < 0.001, *d* = 3.677), 50 mm and 105 mm (mean = 1.803, SD = 0.025) (p < 0.001, *d* = 4.407), and 85 mm and 105 mm (p = 0.016, *d* = 0.627).

### Geometric morphometrics

Using permutational multivariate ANOVA we found statistically significant shape differences in female targets between the faces of the same individuals taken at different focal lengths (F_(1,64)_ = 2.539, p = 0.004, R^2^ = 0.038). The differences between focal length pairs were significant in all three combinations: 50 mm × 85 mm (F_(1,42)_ = 1.814, p < 0.001, R^2^ = 0.041); 50 mm × 105 mm (F_(1,42)_ = 2.463, p < 0.001, R^2^ = 0.055); 85 mm × 105 mm (F_(1,42)_ = 0.41, p < 0.001, R^2^ = 0.01).

**Fig 5:**
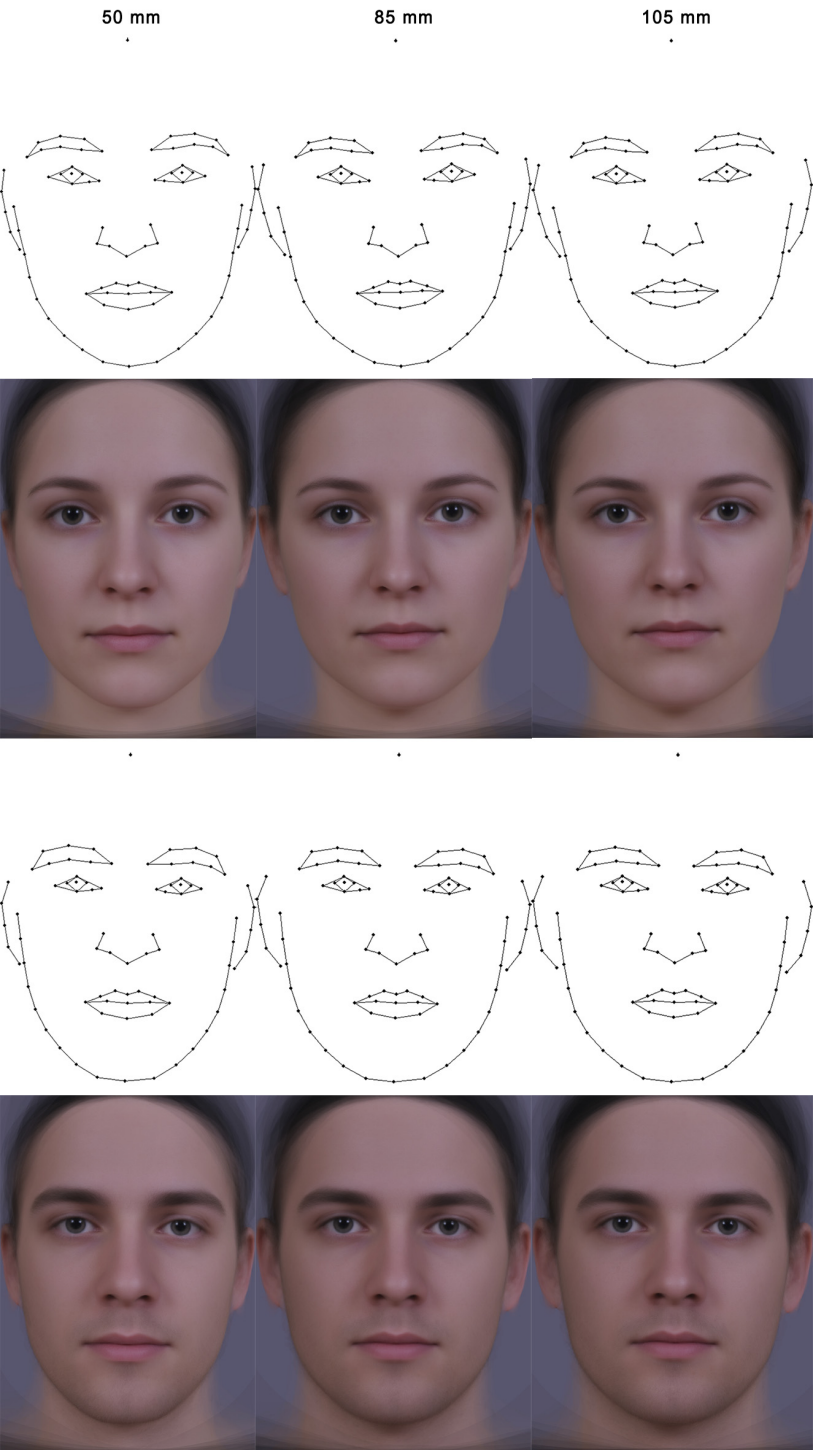
Consensual configurations and composites of female and male images for the 50 mm, 85 mm and 105 mm focal lengths.

A similar pattern was found for the male targets (F_(1,66)_ = 2.765, p < 0.001, R^2^ = 0.04). Subsequent comparison of focal length pairs again showed significant differences for all three combinations: 50 mm × 85 mm (F_(1,44)_ = 1.739, p < 0.001, R^2^ = 0.038); 50 mm × 105 mm (F_(1,44)_ = 2.721, p < 0.001, R^2^ = 0.058); 85 mm × 105 mm (F_(1,44)_ = 0.23, p = 0.007, R^2^ = 0.005) (Fig 5).

## Discussion

The main aim of our study was to test for possible effects of variation in focal length on perceptual judgements and shape distortions of male and female facial photographs. Photographs were taken of the same individual at focal lengths of 50 mm, 85 mm, and 105 mm equivalents. We found that facial photographs taken with 105 mm focal length equivalent were judged by both male and female raters as the most attractive, dominant, and masculine/feminine, irrespective of the target’s sex. In contrast, photographs of both males and females taken with 50 mm focal length equivalent were perceived as the least attractive, dominant, and masculine/feminine. The differences in perception were further supported by our shape analysis. We found that fWHR, as measured from the photographs, significantly varied across focal lengths in both male and female faces; fWHR taken at 50 mm was significantly smaller than the two greater focal lengths (85 mm and 105 mm). Results of the geometric morphometrics analyses similarly showed significant differences in overall facial shape between the focal lengths used in both sexes. In both male and female faces the following pattern was observed for images taken at the 50 mm focal length as compared to the longer focal lengths: overall rounded face, larger and wider set eyes, wider set eyebrows, rounded, longer and broader nose, taller forehead, rounded chin and disappearing ears obscured with cheeks (Fig 3).

The changes in facial dimensions, shape, and facial perception found in our study appear to be a consequence of variation in perspective distortion produced by the different focal lengths. In objects captured with shorter focal lengths, perspective distortion produces an appearance expanded in its depth, which makes faces look rounded and facial traits closer to camera are perceived seemingly bigger (e.g., nose) while more distant traits look smaller (e.g., ears). The faces captured with the shortest focal length appear overall to be rounded due to the vertically oblong shape of the human head, which produces the most noticeable radial distortion at the sides of head. In contrast, objects captured with longer focal lengths look compressed in depth, which makes faces look flatter [32]. The resulting effect shows smaller facial width-to-height ratio for faces captured with shorter focal lengths [19].

For several characteristics (attractiveness in both female and male photographs and femininity in female photographs) the differences between judgements of the facial photographs taken with 85 mm and 105 mm were not significant. This effect might be a result of a smaller range between particular focal lengths and consequently less pronounced perspective distortion. Our analysis of facial shape supports this interpretation, as the observed effect sizes were rather small. Similarly, fWHR measurements of female faces did not significantly differ between photographs taken with 85 mm and 105 mm. Further, male faces have, on average, substantially more distinctive facial traits compared to females (e.g., more pronounced zygomatic arches, eye ridges, broader chin), hence their photographs might be more affected by distortions. Alternatively, the perception of some characteristics might be of greater importance in social interactions than others. Therefore, smaller differences in facial shape might not be reflected in attributed characteristics.

Here we employed a forced-choice paradigm, i.e., raters were simultaneously shown all three photographs of each target and they were asked to order the triads of photographs with respect to a given characteristic (e.g., attractiveness). The advantage of this approach is its higher sensitivity allowing for detection of subtle effects, on the other hand this setting may overestimate the actual effect. Another rating paradigm which is frequently employed in the face research is rating of each targets´ face on Likert scales one by one. However, this approach is somewhat less sensitive in detecting subtle effects. Future studies should therefore test whether a similar pattern as observed in the current study can be generalized to the other rating paradigm.

Apart from the focal length, several other important factors may affect the resulting portrait photographs; including target to camera distance [19,28,31]. As 3D objects (e.g., faces) are captured in form of 2D images on a plain (e.g., camera sensor) via perspective projection, the resulting image changes with a distance from the centre of projection, even when equated for its size [19,31]. The target to camera distance could be set by determining a field of view (or angle of view), which is a function of sensor size, focal length, and target to camera distance. Although there is no general consensus on ideal target to camera distance [28], the results of Bryan et al. [19] show considerable impact of this variable on perceived characteristics. They found that faces captured from a shorter distance (45 cm) were rated as less attractive and less trustworthy as compared to the images taken from a longer distance (135 cm). A possible explanation lays in interpersonal distance as a variable influencing social behaviour [43]; Bryan et al. [19] hypothesized that the distance-dependent perspective projection of a face might serve as a cue for social judgments and faces photographed from within personal space were judged more negatively on certain characteristics. The same principle applies to the distance between the rater and the assessed image. Positioning the rater at a distance from the image that equals the target to camera distance should give an accurate impression of the scene. However, raters are usually not positioned in the centre of projection when viewing the images which may in turn bias their impression [31]. Moreover, it appears that people tend to prefer shorter viewing distances for images captured with longer focal length and longer distances for the images captured with shorter focal lengths [31].

Previous research has further shown that colour representation [24,44,45] and head position [25] influence the resulting image. Specifically, changes in face colour affect perceived health and attractiveness with higher judgements of redder faces [24,44–46] (but see Burriss et al. [23]), while downward head tilt is perceived as more intimidating through the manipulation of fWHR [25]. However, downward tilt also increases attractiveness of female faces in mate choice context [47,48].

In our study we found that certain camera settings, in this case focal length, can considerably influence perception of resulting photographs. Distortion of the facial shape may increase the chance of both type I (falsely positive results) and/or type II (falsely negative results, e.g., floor or ceiling effect) errors. For instance, false positive results might occur by taking one set of the photographs with a certain focal length and another set with a different focal length, therefore introducing systematic error. This may occur in studies using images downloaded from the internet, where the image acquisition settings may vary in systematic fashion. For instance, studies measuring fWHR from downloaded images may reflect systematic differences in image acquisition rather than actual differences in facial proportions (e.g., successful athletes in combat sports might be depicted as tougher by adjusting camera settings compared to their less successful counterparts). In contrast, using a rather wide lens may skew the ratings of some positively perceived characteristics such as trustworthiness or attractiveness to the lower end of the scale, which might obscure the chances of finding an actual effect.

Our results thus indicate that the focal length should be adjusted according to aims of a particular study. Based on our findings and previous work it seems that longer focal lengths (e.g., 85 mm full frame equivalent) produce more positively judged images [30,49] which might be an advantageous approach in attractiveness studies as it diminishes chances of the floor effect. On the other hand, longer focal lengths entail higher demands on target to camera distance to obtain a suitable field of view and accordingly increase space requirements. Selection of an adequate focal length is thus frequently a trade-off between the lens and space available for image acquisition.

In sum, our study provides additional evidence that the methodology of photograph acquisition can influence the results of perceptual studies. We showed that facial photographs taken with various focal lengths differ in their proportions as demonstrated by the fWHR, overall facial shape as analysed by the GMM and perceived characteristics judged by independent set of raters. These results highlight the importance of adopting a standardized methodology for photograph acquisition, at least within each particular study. This is often not possible in the case of photographs downloaded from online sources. Interestingly, although this approach is methodologically questionable, it is an increasingly popular method within various branches of psychological science due to its easy availability. Further, we urge researchers capturing images in the lab to report details of image acquisition parameters and settings such as camera brand and type, sensor size (crop factor), lens and focal length, exposure parameters (F-stop, shutter speed and ISO), light source(s), modifier(s), and scene setup (e.g., distance from photographed target). Providing such details would enable a more rigorous analysis of the discrepancies between individual studies–a standard that is currently not yet achieved even in some prestigious academic journals.

## Supporting Information

S1 Dataset Data from ratings and fWHR measurements (.XLSX). Data on GMM are available from the authors.
(XLSX)
